# Analysis of living habit risk factors for esophageal cancer in central China: A bi-center case-control study

**DOI:** 10.3389/fonc.2023.1077598

**Published:** 2023-01-24

**Authors:** Lingzhi Yuan, Peijun Shen, Shaopeng Zheng, Dongwen Wu, Xinmeng Li, Ting Cai, Yao Yao, Yunhe Song, Fen Wang

**Affiliations:** ^1^ Department of Gastroenterology, The Third Xiangya Hospital, Central South University, Changsha, Hunan, China; ^2^ Hunan Key Laboratory of Non-resolving Inflammation and Cancer, Central South University, Changsha, Hunan, China; ^3^ Department of Gastroenterology, The First Affiliated Hospital of Xinxiang Medical University, Xinxiang, Henan, China; ^4^ Department of thoracic surgery, Linzhou Esophageal Cancer Hospital, Linzhou, Henan, China

**Keywords:** lifestyle, dietary habits, esophageal cancer, risk factors, therapeutics

## Abstract

**Background:**

Esophageal cancer remains a public health problem in many countries, especially developing countries. The early lifestyle preventive measures mentioned in the treatment guidelines for esophageal cancer are very limited. We aimed to evaluate the risk factors for esophageal cancer in a high-incidence area in China and to provide evidence for clinical intervention in esophageal cancer prevention.

**Methods:**

Symptom and lifestyle/habit questionnaires including 19 items were designed. The correlation between the occurrence of esophageal cancer and living habits was analyzed retrospectively through questionnaire survey. A total of 708 subjects (365 esophageal cancer, 343 non-esophageal cancer) enrolled from two hospitals in central China (Linzhou Esophageal Cancer Hospital and The Third Xiangya Hospital of Central South University) completed symptom and lifestyle/habit questionnaires. We used conditional logistic regression to estimate the odds ratio (OR) with consideration of 95% confidence interval (CI).

**Results:**

The composition ratio analysis showed that the top five lifestyle factors related to esophageal cancer were eating too fast, drinking, hot drinks, smoking and overeating. Univariate analysis showed that 15 factors, including male sex, smoking, drinking, eating too fast, overeating, hot drinks, greasy food, acidic food, hard food, strong tea, coffee, bedtime immediately after meals, eating food before bedtime, difficult defecation, and an overtight belt, were associated with esophageal cancer (all P <0.05). Logistic multivariate regression analysis showed, drinking (OR 3.609, 95%CI 2.223-5.859; P=0.000); hot drinks (OR 2.672, 95%CI 1.786-3.997; P=0.000); overeating (OR 2.110, 95%CI 1.411-3.154; P=0.000); eating too fast (OR 1.879, 95%CI 1.274-2.772; P=0.001); strong tea (OR 1.882, 95%CI 1.171~3.023; P=0.009); hard food (OR 1.723, 95%CI 1.113-2.667; P=0.015); smoking (OR 1.686, 95%CI 1.045-2.720; P=0.032), which were significantly associated with the development of esophageal cancer.

**Conclusion:**

The unhealthy lifestyles of patients in high-incidence areas of esophageal cancer in central China are significantly associated with the incidence of esophageal cancer. Lifestyle changes that address these factors, especially overeating and eating too fast, which are rarely studied or discussed despite being common, may improve esophageal cancer management and treatment outcomes. The present results may be used as a reference for preventive education and treatment.

## Introduction

Esophageal cancer remains a public health problem in many countries, especially developing countries. According to the latest statistical data (Glbocan2020), esophageal cancer ranks seventh in terms of incidence (604,000 new cases) and sixth in mortality overall (544,000 deaths). Eastern Asia exhibits the highest regional incidence rates, in part due to the high burden in China (1). The burden of esophageal cancer in China is serious: in 2019, there were 278,121 new cases and 257,316 deaths by esophageal cancer in China, accounting for more than half of the number of cases and deaths in the world (2). The age-standardized incidence rate (ASIR), age-standardized death rate (ASDR), and age-standardized disability adjusted life-years (DALYs) rate of esophageal cancer in China were about 1.3 times that of East Asia and Pacific, twice that of the world, and more than twice that of Japan and South Korea (3). Notably, the geographic distribution of esophageal cancer varied greatly in China. The incidence rate and mortality of esophageal cancer is the highest in central China (4). Surprisingly, the incidence rate of esophageal cancer in rural areas of central China was more than four times that in urban areas of northeast China (4). Linxian County in Henan Province, Cixian County in Hebei Province, and Yangcheng County in Shanxi Province are regions with high incidence rate and mortality of esophageal cancer in China and even in the world. In particular, Linzhou city lies at the junction of Henan, Shanxi, and Hebei, which is one of the representative areas with the highest incidence of esophageal cancer in China and is the first base for the early diagnosis and early treatment of esophageal cancer. Linzhou city started to organize and implement the following five preventive measures in 1972: use of anti-mildew measures, use of deamine, change bad eating habits (do not eat persimmon bran contaminated with mold and nitrosamines), stop eating pickled vegetables, and stop eating moldy food (5). After modifying the unhealthy living environment and diet, the incidence of esophageal cancer (squamous carcinoma) showed a downward trend, and the incidence of adenocarcinoma did not increase (6). Moreover, the patient survival rates gradually improved. However, there is still a large disease burden of esophageal cancer (6); by the time symptoms of esophageal cancer appear, the cancer is often at an advanced stage and has a poor prognosis, emphasizing the significance of early prevention to lower the burden of esophageal cancer.In fact, the improvement in prognosis is likely to be partly explained by better awareness of esophageal cancer and its risk factors among physicians and individuals.

The geographic variation in esophageal cancer occurrence in China strongly suggests that environmental or lifestyle factors are major contributors to the etiology of esophageal cancer. The risk factors of esophageal cancer are discrepant in different countries and regions. Among all potentially modifiable risk factors quantified in the Global Burden of Disease (GBD) 2019, the deaths of esophageal cancer worldwide in 2019 were primarily attributable to smoking, followed by alcohol use, high BMI, diet low in fruits and diet low in vegetables (7). However, the changes of the attributable burden to these risk factors during the study period were heterogeneous. For example, a large randomised intervention trial with 26 years of follow-up in a nutrient-deficient population in China found no benefit of multivitamins supplementation in reducing esophageal cancer-specific mortality (8). A nationwide Swedish cohort study comparing 34,437 patients who underwent obesity surgery and 123,695 obese individuals who did not receive such surgery found no clear reduction in OAC risk associated with obesity surgery. Hence, it is necessary to further identify and analyze other potential risk factors affecting the occurrence and development of esophageal cancer (9).

Notwithstanding, the early lifestyle preventive measures mentioned in the treatment guidelines for esophageal cancer are limited. Our previous study based on data from multiple centers in China demonstrated that the incidence of gastroesophageal reflux disease (GERD) is also closely correlated with unhealthy lifestyle habits, including eating too fast, eating hot food, and overeating (10). The current evidence suggests that gastroesophageal reflux disease (GERD) is closely associated with the prevalence of esophageal cancer (11). We speculate that the above-mentioned GERD related risk factors may also be closely related to the occurrence and development of esophageal cancer. Identifying the risk factors for esophageal cancer and promoting accurate prevention methods are crucial to alleviating the global burden of esophageal cancer. The present study investigated the unhealthy living habits of patients in high-risk areas for the occurrence of esophageal cancer, providing a reference for the clinical prevention of esophageal cancer by improving unhealthy living habits.

## Methods

### Research objects

In total, 365 patients with esophageal squamous cell carcinoma/adenocarcinoma confirmed by pathological examination at Linzhou City (Lin County) Esophageal Cancer Hospital in Henan Province and The Third Xiangya Hospital of Central South University from January 2019 to January 2021 were selected as the case group, and 343 cases of superficial gastritis screened by gastroscopy in the same two hospitals during the same period were selected as the control group. Inclusion criteria: residents who were permanent residents of the region and have lived for more than 6 months, clinical data were complete. Exclusion criteria: suffering from severe mental illness, communication disorders, other chronic diseases, or severe comorbidities with other malignancies; suffering from other upper gastrointestinal lesions (such as gastric ulcers, duodenal ulcers, and gastric polyps); with severe cardiopulmonary and other organ failure and blood systemic disease; could not complete the questionnaire; with incomplete clinical data. Written informed consent forms were obtained from all participants before questionnaire distribution.

### Research methods

Data on the living and eating habits of eligible individuals were collected by questionnaires. After trained doctors completed the data collection, the quality control personnel checked the form, checked the information, saved the information, and entered the information. The quality control personnel also randomly checked a certain proportion of the respondents by telephone inquiry and review, and they repeatedly verified the consistency of the investigation. In this survey, 30 subjects were selected for the second survey, and eating habits, such as smoking, drinking, tea drinking, family history of esophageal cancer, and food intake, were evaluated. The results showed that the kappa value of the survey index was greater than 0.75, which indicated reliable data information for the survey.

### Questionnaire survey

Self-designed dietary survey questionnaires were designed based on well-accepted food frequency questionnaires (FFQs) (12) and the living and eating habits of Chinese Populations. The FFQs was used to investigate the daily, weekly, monthly or annual intake frequency of each food and the average amount of each food consumed in the past year. It has been widely used internationally. The trained investigators performed a face-to-face survey using standard questionnaires, and participants answered the questions by themselves. The content of the questionnaire included basic information of patients (gender and age), symptoms within four weeks before onset (early symptoms, including reflux, acid reflux, heartburn, retrosternal pain, retrosternal pain, and foreign body sensation in the throat), preonset eating habits and preonset living habits.

### Assessment of lifestyle and eating habits

The following 11 eating habits were considered: eating too fast (eat less than 10 minutes per meal and chew less than 10 times per bite), overeating (having satiety after eating and continuing to eat until you cannot eat another bite), eating too hot (food temperature exceeding 60°C), preference for soup, preference for spicy food (such as onion, garlic, ginger, chili, Sichuan pepper, and pepper), preference for high-fat food (such as chocolate, fried food, fatty meat, animal offal, and cream products), preference for acidic foods (such as pickles, acid drinks, oranges, and lemons), preference for sweet food (such as cream, cakes, and chocolates), preference for hard foods (such as walnuts and peanuts), preference for strong tea (amount of tea more than one cup of 3-4 g of tea soup), and preference for coffee. The lifestyle habits included the following 8 items: drinking; smoking; bedtime immediately after meals (bedtime within 30 minutes after eating); eating food before bedtime (eating within 2 hours of bedtime); difficult defecation (weak and time-consuming defecation); dyscoimesis (excessive or insufficient sleep, sleepwalking, night terrors, and nightmares); nervousness and anxiety (vexation, irritability, panic, and sorrow); and overtight belt.

### Quality control

Research subjects were checked and determined by professionals with extensive clinical experience. The questionnaire was completed by uniformly trained physicians after consultation with experts. In order to decrease the memory bias, the interviewee should be patients himself or his closest relative. The interviewers should be trained professionally, understand content of the questionnaire and natives living habits, know some of the common idiom, and also should learn to communicate with the local residents. The questionnaire with poor quality must be investigated once again. A special quality control staff member checked the completed forms. After confirming that there were no errors, the staff member saved the completed forms and assigned them into groups.

### Statistical analysis

EXCEL was used for data entry to ensure the accuracy of the data. SPSS 25.0 statistical software was used for data processing and analysis. Continuous variables were expressed as mean ± sd and analyzed by a t test. Categorical variables were analyzed by a χ^2^ test. Univariate analysis was first performed on the relevant factors of the incidence of esophageal cancer, <0.05 was considered statistically significant. Potential esophageal cancer risk factors with a *P* value < 0.05 in the univariable analysis were included in the esophageal cancer multivariable analysis using logistic regression. During logistic regression analysis, we chose to adjust all confounding factors for age (Numerous studies have reported increased risk of esophageal cancer associated with age. While, age has no significance in the univariate analysis of our data). After adjusting for confounders, the strength of the association was quantified as odds ratios (ORs), and 95% confidence intervals (CIs) around the OR were used to quantify precision.

## Results

### Characteristics of research subjects

**Figure 1:**
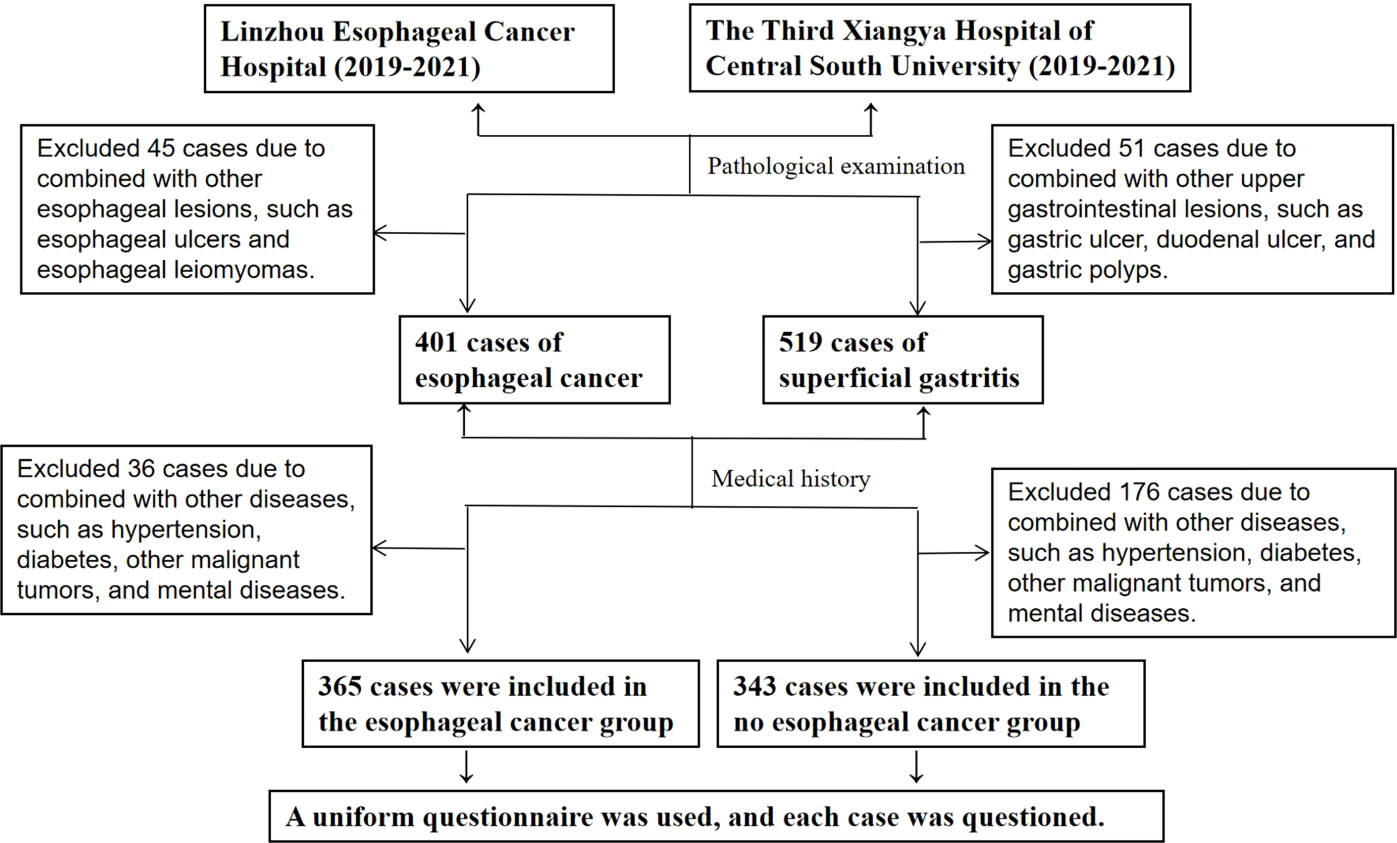
Study recruitment flowchart. In total, 365 patients with esophageal cancer confirmed by pathological examination at Linzhou City (Lin County) Esophageal Cancer Hospital in Henan Province and The Third Xiangya Hospital of Central South University from January 2019 to January 2021 were selected as the case group, and 343 cases of superficial gastritis screened by gastroscopy and pathological examination in the same two hospitals during the same period were selected as the control group.

The enrollment and group designations are summarized in **Figure 1**. **Table 1** shows the following basic demographic characteristics of the two groups: 1) case group, which consisted of 365 cases of esophageal cancer, 257 cases of male patients (accounting for 70.4%), 108 cases of female patients (accounting for 29.6%), mean age 61.75 ± 9.064 years, and normal distribution of ages; and 2) control group, which consisted of 343 cases of gastritis, 151 cases of male patients (accounting for 44%), 192 cases of female patients (accounting for 56%), mean age 57.37 ± 13.325 years, and normal distribute of the ages were in a normal distribution. There was no significant difference between the two groups in terms of average age. Univariate analysis with the χ^2^ test indicated that male sex was significantly associated with the presence of esophageal cancer.

**Table 1:** Univariate analysis of esophageal cancer-related risk factors (demographic factors).

variate		Esophageal cancer n (%)	Gastritis n (%)	*X^2^*	*P*
Gender	Male	257 (70.4)	151 (44.0)	50.424	0.000***
	Female	108 (29.6)	192 (56.0)		
Age	mean ± SD (years)	61.75 ± 9.064	57.37± 13.325		
	<60	155 (42.5)	141 (41.1)	0.134	0.714
	≥60	210 (57.5)	202 (58.9)		

*** indicates a significant statistical difference between the esophageal cancer group and the non-esophageal cancer group.(P<0.001).

### Percentage of esophageal cancer early symptoms

When the incidence of early symptoms in patients with esophageal cancer was analyzed using the composition ratio of the primary symptoms in the case group, the following symptoms in descending order were found: swallowing obstruction (72.78%), reflux (51.51%), pharyngeal foreign body sensation (46.03%), acid reflux (44.11%), postprandial fullness (43.29%), retrosternal pain (43.56%), heartburn (38.90%), and subxiphoid burning (38.08%).

### Esophageal cancer-correlated factors

**Figure 2:**
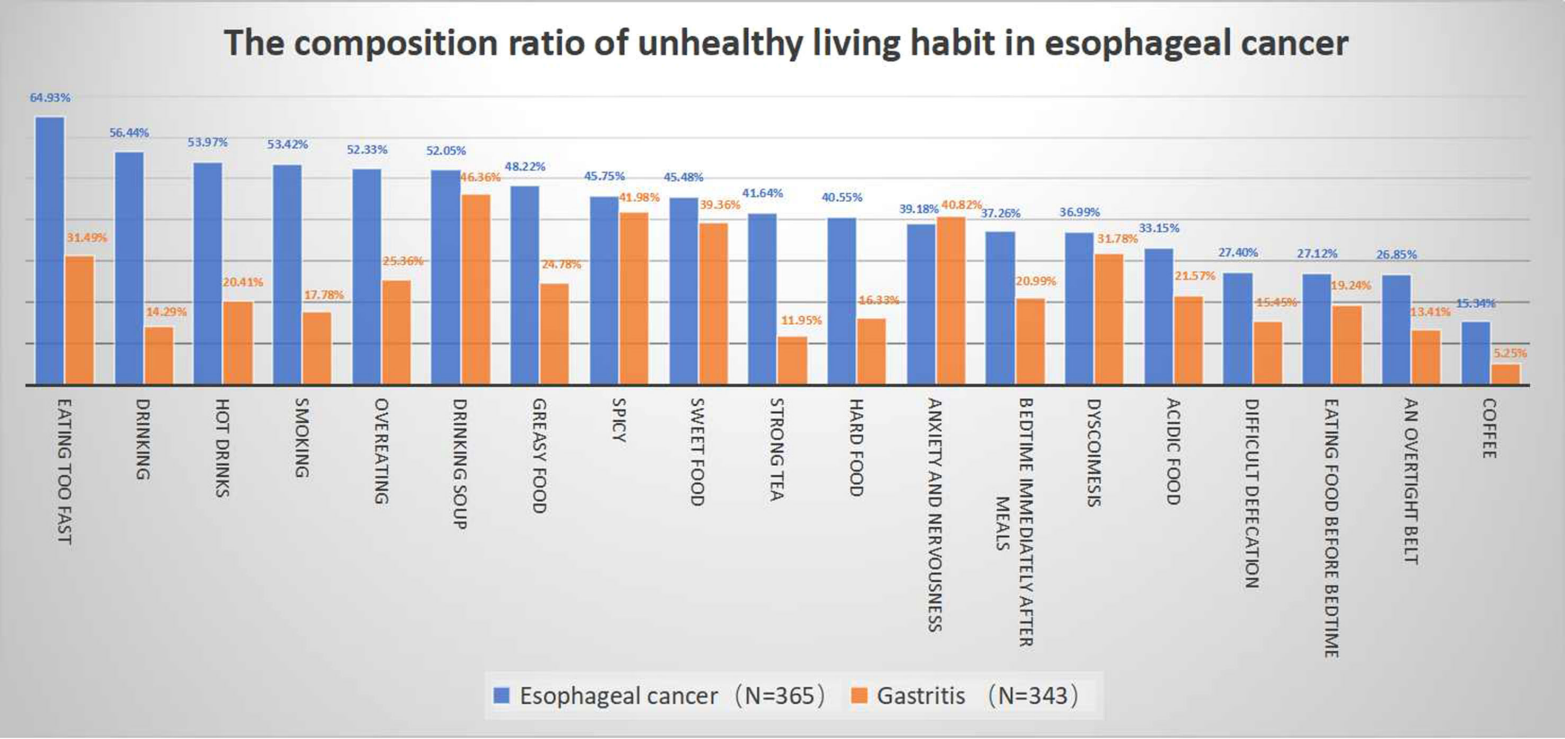
The composition ratio of unhealthy living habit in esophageal cancer.

The composition ratio analysis results for esophageal cancer-correlated factors are shown in **Figure 2**. Eating too fast, drinking, hot drinks, smoking, and overeating were the habits shared by the majority of the esophageal cancer patient group.

As shown in **Table 2**, univariate analysis indicated a correlation of esophageal cancer with smoking, drinking, eating too fast, overeating, hot drinks, greasy food, acidic food, hard food, strong tea, coffee, bedtime immediately after meals, eating food before bedtime, difficult defecation, and an overtight belt (all P <0.05). As shown in **Table 3**, logistic regression analysis of the habits identified to be related to esophageal cancer in the univariate analysis implicated the following habits as risk factors (in descending order): drinking (OR 3.609, 95%CI 2.223-5.859; P=0.000); hot drinks (OR 2.672, 95%CI 1.786-3.997; P=0.000); overeating (OR 2.110, 95%CI 1.411-3.154; P=0.000); eating too fast (OR 1.879, 95%CI 1.274-2.772; P=0.001); strong tea (OR 1.882, 95%CI 1.171~3.023; P=0.009); hard food (OR 1.723, 95%CI 1.113-2.667; P=0.015); and smoking (OR 1.686, 95%CI 1.045-2.720; P=0.032).

**Table 2:** Univariate analysis of unhealthy living habit for esophageal cancer.

variate	esophageal cancer group		non-esophageal cancer group		*X* ^2^	*P*
	NO n (%)	YES n (%)	NO n (%)	YES n (%)		
Smoking	170 (46.6)	195 (53.4)	282 (82.2)	61 (17.8)	97.303	0.000***
Drinking	159 (43.6)	206 (56.4)	294 (85.7)	49 (14.3)	136.343	0.000***
Eating too fast	128 (35.1)	237 (64.9)	235 (68.5)	108 (31.5)	79.168	0.000***
Overeating	174 (47.7)	191 (52.3)	256 (74.6)	87 (25.4)	53.912	0.000***
Hot drinks	168 (46.0)	197 (54.0)	273 (79.6)	70 (20.4)	84.807	0.000***
Drinking soup	175 (47.9)	190 (52.1)	184 (53.6)	159 (46.4)	2.298	0.130
Spicy	198 (54.2)	167 (45.8)	199 (58.0)	144 (42.0)	1.021	0.312
Greasy food	189 (51.8)	176 (48.2)	258 (75.2)	85 (24.8)	41.736	0.000***
Acidic food	244 (66.8)	121 (33.2)	269 (78.4)	74 (21.6)	11.874	0.000***
Sweet food	199 (54.5)	166 (45.5)	208 (60.6)	135 (39.4)	2.711	0.100
Hard food	217 (59.5)	148 (40.5)	287 (83.7)	56 (16.3)	50.578	0.000***
Strong tea	213 (58.4)	152 (41.6)	302 (88.0)	41 (12.0)	78.612	0.000***
Coffee	309 (84.7)	56 (15.3)	325 (94.8)	18 (5.2)	19.252	0.000***
Bedtime immediately after meals	229 (62.7)	136 (37.3)	271 (79)	72 (21)	22.558	0.000***
Eating food before bedtime	266 (72.9)	99 (27.1)	277 (80.8)	66 (19.2)	6.145	0.013*
Difficult defecation	265 (72.6)	100 (27.4)	290 (84.5)	53 (15.5)	14.895	0.000***
Dyscoimesis	230 (63)	135 (37)	234 (68.2)	109 (31.8)	2.123	0.145
Anxiety and nervousness	222 (60.8)	143 (39.2)	203 (59.2)	140 (40.8)	0.198	0.657
An overtight belt	267 (73.2)	98 (26.8)	297 (86.6)	46 (13.4)	19.709	0.000***

*,*** indicates that the esophageal cancer group and the non-esophageal cancer group have significant differences at the levels of P <0.05 and P <0.001, respectively.

**Table 3:** Multivariate analysis of lifestyle risk factors for esophageal cancer.

Factor	β	S.E.	Wals χ2	P	OR	95% CI of OR*
Gender	0.287	0.220	1.705	0.192	1.332	0.866~2.050
Smoking	0.523	0.244	4.588	0.032*	1.686	1.045~2.720
Drinking	1.284	0.247	26.953	0.000***	3.609	2.223~5.859
Eating too fast	0.631	0.198	10.136	0.001**	1.879	1.274~2.772
Overeating	0.747	0.205	13.249	0.000***	2.110	1.411~3.154
Hot drinks	0.983	0.206	22.846	0.000***	2.672	1.786~3.997
Greasy	0.182	0.217	0.700	0.403	1.199	0.784~1.835
Acidic food	0.061	0.224	0.073	0.787	1.062	0.685~1.647
Hard food	0.544	0.223	5.959	0.015*	1.723	1.113~2.667
Strong tea	0.632	0.242	6.829	0.009*	1.882	1.171~3.023
Coffee	0.286	0.349	0.673	0.412	1.331	0.672~2.638
Bedtime immediately after meals	0.221	0.218	1.023	0.312	1.247	0.813~1.914
Eating food before bedtime	-0.173	0.241	0.515	0.473	0.841	0.525~1.349
Difficult defecation	0.364	0.245	2.207	0.137	1.439	0.890~2.324
An overtight belt	0.413	0.254	2.645	0.104	1.511	0.919~2.486

*,**,*** indicates that the esophageal cancer group and the non-esophageal cancer group have significant differences at the levels of P <0.05, P <0.01, and P <0.001, respectively. *Adjusted for age (continuous).

## Discussion

In the present study, we found that the top lifestyle factors favoring esophageal cancer were eating too fast, drinking, hot drinks, smoking, and overeating. Logistic multiple regression analysis implicated drinking, hot drinks, overeating, eating too fast, strong tea, hard food, and smoking as contributors to esophageal cancer.

In contrast to the rising trend of esophageal adenocarcinoma in Western countries in recent years, squamous cell carcinoma continues to be dominant in esophageal cancer in Asian and Middle Eastern countries, such as China and Japan (13, 14). Although the specific pathogenesis of esophageal cancer is currently unclear, many factors are linked to its occurrence. Some studies have demonstrated that esophageal cancer is relevant to familial heredity, which may be explained by the presence of chromosome mutations and increased brittleness in the genetic process. Decreased DNA repair capacity leads to increased susceptibility to genetic predisposition (15). Previous studies have proposed that eating onion garlic, fresh fruits, and soy products reduces the risk of esophageal cancer, probably *via* antioxidants inhibiting the conversion of nitrate into nitrite in the body, thereby reducing the endogenous damage to DNA and enhancing mucosal recovery ability (16). Poor diet and lifestyle have induced or aggravated the genesis and progression of esophageal cancer. Furthermore, many studies have confirmed that there is a causal relationship of the consumption of tobacco and alcohol with the incidence of esophageal cancer. A case–control study involving 150 million people in 103 regions of China has suggested that smoking is an important risk factor for esophageal cancer (17). A report of the World Cancer Research Fund (WCRF) has conclusively established that alcohol consumption is linked to the incidence of esophageal cancer, indicating that increased alcohol consumption results in an increase in esophageal cancer occurrence. In line with the preceding findings, the results of the present study suggested that smokers and drinkers are more likely to develop esophageal cancer.

Our previous study discovered that unhealthy eating habits of sweet food, strong tea, and a greasy diet are related risk factors for the onset of GERD (10). Moreover, GERD can lead to an increased incidence of esophageal adenocarcinoma (11), and the present survey suggested that these poor living habits also cause an increase in the incidence of esophageal cancer. Eating these types of food lowers the pressure of the esophageal sphincter, stimulates gastric mucosa nerve ending receptors to accelerate gastric secretion, aggravates inflammation of the esophageal mucosa, and delays gastric emptying (18). Several studies have found that drinking hot tea increases the incidence of esophageal cancer, but some have suggested that this phenomenon is attributed to the temperature over 60 degrees when drinking tea (19). An animal model of esophageal squamous cell carcinoma has concluded that overheated water increases the carcinogenic effect of chemicals (20). In the present study, we also observed that an overtight belt was a relevant risk factor for esophageal cancer, which may be due to elevated abdominal pressure leading to dry stools, difficult defecation, and aggravation of gastroesophageal reflux. Increased abdominal pressure is likely to cause hiatal hernias (21), which may also contribute to esophagitis and further to esophageal cancer.

*The most striking findings in the present study were two factors correlated with esophageal cancer, namely, eating too fast and overeating.* Currently, there are limited studies examining these two factors. Consistent with previous studies, the present study further showed that eating hot food was associated with the incidence of esophageal cancer. Eating too fast or eating hard food often results in food not being fully ground, causing food residues entering the esophagus to be too large in volume and rough on the surface. Thus, inadequate chewing of food may damage the mucosal lining of the esophagus and cause cell proliferation. Eating too fast often leads to a large amount of food intake in a short period of time, thus causing excessive pressure on the stomach, delaying gastric emptying, and stimulating excessive gastric acid secretion, which induces a large amount of gastric content to flow back into the esophagus through both mechanical and physiological factors. Eating too fast easily contributes to obesity, and a combination of multiple risk factors accelerates disease progression. Eating food that is too hot directly damages the esophageal mucosal barrier. Domestic and overseas studies have shown that hot food or hot drinks can increase the risk of esophageal cancer (22). Eating hot food or drinking hot drinks over a long-term and recurrent period will result in long-term and repeated diffuse interstitial inflammation of the esophagus, promoting atypical hyperplasia of the mucosa epithelium, and squamous dysplasia is closely related to the risk of esophageal squamous cell carcinoma (23).

However, our research has some limitations. Firstly, the recall and selection are the common biases in all such studies, however, we tried our best to avoid or reduce these biases, but still these can be considered as limitation of our study. Secondly, some influencing factors were widely reported, but were not included in the present study. For example, GERD was established as a strong and dose-dependent risk factor for esophageal cancer in the late 1990s (24). Barrett’s esophagus, a metaplastic change in the esophageal lining from normal squamous to a specialised columnar epithelium, has since long been recognised as a precursor lesion to esophageal cancer in individuals with chronic GERD (25). However, whether antireflux therapies reduce the risk of esophageal cancer associated with GERD is a matter of debate (26, 27). Studies have shown that infection with the gastric bacterium *Helicobacter pylori* (*H. pylori)* has been associated with a 40-60% reduced risk of esophageal cancer (28, 29). Recent meta-analysis shows that *H. pylori* infection may reduce the risk of esophageal adenocarcinoma, but such “protection effect” may be overestimated (30). The relevant risk factors in the present study are only preliminary conclusions. We are currently improving our research methods and incorporating more influencing factors into the national multicenter cohort study for analysis. Esophageal cancer is a deadly disease primarily affecting developing countries. More research efforts should be made for a better understanding of the aetiology of esophageal cancer to support evidence-based prevention of this deadly cancer in both the developed and developing parts of the world.

In conclusion, the risk factors for esophageal cancer include drinking, hot drinks, overeating, eating too fast, strong tea, hard food, and smoking. Lifestyle changes that address these factors, especially overeating and eating too fast, which are rarely studied or discussed despite being common, may improve esophageal cancer management and treatment outcomes. Therefore, lifestyle habits, such as eating slowly, eating several small meals rather than a full meal, avoiding strong tea, avoiding hard food, advocating a warm diet, quitting smoking, and quitting drinking, may effectively prevent and reduce the incidence of esophageal cancer.

## Data availability statement

The original contributions presented in the study are included in the article/supplementary material. Further inquiries can be directed to the corresponding author.

## Ethics statement

The studies involving human participants were reviewed and approved by the Xiangya Third Hospital Ethics Committee of Central South University. The patients/participants provided their written informed consent to participate in this study.

## Author contributions

LY wrote the main manuscript text. PS collected data. SZ and DW participated in the data analysis. XL, TC and YY performed the statistical analysis, interpretation of the data, and statistical expertise. YS and FW contributed in the review of final edition of the manuscript. FW carried out the design of the study. All authors contributed to the article and approved the submitted version.
